# Editorial: Exercise physiology and its role in chronic disease prevention and treatment—mechanisms and insights

**DOI:** 10.3389/fphys.2022.1038119

**Published:** 2022-09-30

**Authors:** Hassane Zouhal, Urs Granacher, Anthony. C. Hackney, Shunchang Li, Ismail Laher

**Affiliations:** ^1^ M2S (Laboratoire Mouvement, Sport, Santé), University Rennes, Rennes, France; ^2^ Insrtitut International des Sciences du Sport (2I2S), Irodouer, France; ^3^ Department of Sport and Sport Science, Exercise and Human Movement Science, University of Freiburg, Freiburg, Germany; ^4^ Department of Exercise and Sport Science, Department of Nutrition, University of North Carolina, Chapel Hill, NC, United States; ^5^ Institute of Sports Medicine and Health, Chengdu Sport University, Chengdu, China; ^6^ Department of Anesthesiology, Pharmacology& Therapeutics, University of British Columbia, Vancouver, BC, Canada

**Keywords:** physical activity, exercise, training, chronic diseases, health

## Introduction

The performance of regular physical exercise offers protection against all-cause morbidity and mortality, and prevents t atherosclerosis, type 2 diabetes, colon, and breast cancer (Eyre et al., 2004). In addition, exercise training is effective in the treatment of patients with ischemic heart disease, heart failure, and chronic obstructive pulmonary disease (Borghi-Silva et al., 2021). Moreover, sufficient physical activity according to the World Health Organization (150-300 min per week) has several benefits on psychiatric conditions/diseases (depression, anxiety, stress, schizophrenia) and neurological diseases (dementia, Parkinson’s disease, multiple sclerosis) (Pedersen & Saltin, 2015). Evidence-based treatment is the preferred therapeutic approach and provides the most effective management strategy entailing the fewest side effects or risks. Hence, physical exercise is considered a part of a healthy lifestyle, and an important extension of medical treatment and health care (Warburton et al., 2006).

Little is known about the psycho-physiological mechanisms involved in the health benefits of physical exercise, including a lack of knowledge on the effective dosage of programming parameters according to the FITT principle (frequency, intensity, time and type of exercise) (Pedersen & Saltin, 2015; Saeidi et al., 2021; Zouhal et al., 2021; Hortobágyi et al., 2022). As such, further research is needed to determine which exercise type is more effective for the treatment of various chronic diseases and to explore the underlying physiological mechanisms of exercise in chronic diseases such as psychiatric diseases (depression, anxiety, stress, schizophrenia), neurological diseases (dementia, Parkinson’s disease, multiple sclerosis), metabolic diseases (obesity, hyperlipidemia, metabolic syndrome, polycystic ovarian syndrome, type 2 diabetes, type 1 diabetes), cardiovascular diseases (hypertension, coronary heart disease, heart failure, cerebral apoplexy, and claudication intermittent), pulmonary diseases (chronic obstructive pulmonary disease, asthma, cystic fibrosis), musculoskeletal disorders (osteoarthritis, osteoporosis, back pain, rheumatoid arthritis), and several types of cancers. In an attempt to clarify the effects of physical exercise on chronic diseases, the Frontiers Research Topic entitled “Exercise Physiology and its Role in Chronic Disease Prevention and Treatment - Mechanisms and Insights” was launched. Accordingly, the aim of this Research Topic was to provide evidence in the form of original research and or review articles on the protective effects of physical exercise on the treatment of chronic diseases in humans and animals.

## Summary of selected articles from this research topic

Twenty-nine manuscripts were received for this Frontiers Research Topic. After rigorous review, 16 articles were finally accepted for publication. The contributing 80 authors were from 13 countries and four continents, including Canada, China, France, Germany, India, Iran, Japan, Lebanon, Portugal, Tunisia, Turkey, United Kingdom, United States of America. This Research Topic received more than 38,000 views and downloads as of August 2022. The key contents and findings of each paper are as follows:

The study by Gomarasca and colleagues examined the benefits of Nordic exercise (also known as pole walking), which combines moderate levels of cardiovascular exercise with increased muscle activity of the arms, legs, and upper body, on whole body inflammation in elderly women (mean age: 68 years old). Blood levels of inflammatory markers were measured using RT-qPCR before and after 12 weeks of Nordic exercise. Nordic exercise caused modest reductions in the resting levels of the expression of inflammatory markers in both normal and overweight elderly women.

The literature review by Wu et al. summarizes the potential benefits of exercise on cardiovascular, renal, neurological, and pulmonary function in sepsis. The findings, largely based on experimental models of sepsis, suggest that exercise improves outcomes in sepsis by augmenting mitochondrial quality and biogenesis, attenuating inflammation, recovering redox balance, and restoring the health of the gut microbiome.

The potential effectiveness of yoga therapy in managing type 2 diabetes was explored in a systematic review of 13 studies (1,335 patients) by Chen et al. There were significant benefits of short-term yoga (10-24 weeks) on HbA1c, fasting blood glucose, postprandial blood glucose, total cholesterol, triglyceride, and BMI levels, suggesting that yoga may be a useful adjuvant therapy in the usual clinical management of uncomplicated type 2 diabetes.

Wang and colleagues examined the potential benefits of low-intensity aerobic exercise in high-fat diet-associated pulmonary fibrosis in C57BL/6 mice. Aerobic exercise improved obesity-related pulmonary fibrosis, chronic inflammation, and insulin resistance following 16 weeks of a high-fat or chow diet followed by 8 weeks of exercise training.

The narrative review by Zeng et al. summarized symptoms related to knee osteoarthritis and the therapeutic benefits of different exercise types (e.g., aerobic, resistance, neuromuscular training) on patients with knee osteoarthritis. The reported outcomes are specific to the exercise type under investigation. Accordingly, individualized exercise prescriptions are needed.

The study by Li and colleagues investigated the dosage effects of single bouts of high-intensity interval training on brain-derived neurotrophic factor (BDNF) and vascular endothelial growth factor-A (VEGF-A) and cognitive function in healthy young men. Twenty minutes of exercise improved BDNF and VEGF-A as well as cognitive function.

Saedi and colleagues examined the differential effects of high-intensity interval training (HIIT), circuit resistance training (CRT), and moderate-intensity continuous training (MICT) on neuregulin 4 in young men with obesity. HIIT and CRT protocols had greater benefits on blood neuregulin 4 levels, metabolic and cardiovascular risk factors, and body composition.

The systematic review and meta-analysis by Zouhal and colleagues evaluated the influence of exercise training on bone health indices in obese individuals. Their findings, based on 10 studies (889 initial records, 8 countries, 263 participants), suggest that physical exercise has little to no effect on the whole-body bone mineral density in individuals with overweight/obesity.

The narrative review by Razi and colleagues summarized the neuro-invasive properties of SARS-CoV-2 and the possible pathways for the entry of the virus into the central nervous system, and discussed the multimodal effects of exercise on peripheral and central inflammation, blood-brain barrier integrity, glial and neural cells, and remyelination. Moderate exercise training produced health benefits in patients with multiple sclerosis, prior to or after infection with SARS-CoV-2.

Yu et al. examined the effects of exercise on hepatic ApoA5 expression of ApoA5 and TLR4-mediated pathway in mice with high-fat diet (HFD)-induced non-alcoholic steatohepatitis (NASH). Their results demonstrated that exercise improved HFD-induced NASH by triggering the inhibitory effects of the ApoA5 on the TLR4-mediated NF-κB pathway.

A meta-analysis by You et al. reported the effects of different intensities and durations of aerobic exercise on vascular endothelial function of middle-aged and elderly people. Nine studies involving 221 participants were utilized. Vigorous-intensity aerobic exercise (≥8 weeks) improved endothelial function in healthy middle-aged and elderly people.

The study by Feng et al. explored the effects of 4 weeks of hypoxic training on β-aminoisobutyric acid (BAIBA) secretion and white fat browning in inguinal fat in obese rats. Their results showed that this kind of training reduced body weight, Lee’s index, and regulated blood lipid profile. Moreover, hypoxic training up-regulated BAIBA concentrations in gastrocnemius muscle and the circulation, with increased expression of PPARα and UCP-1 in inguinal fat of obese rats and greater white fat browning. The authors concluded that BAIBA could improve the blood lipid profile and stimulate white fat browning by modulating PPARα and UCP-1 expression.

Paillard examined the potential role of using percutaneous electrical stimulation for reconditioning functional capabilities in older subjects utilizing two modalities of electrical stimulation: neuromuscular electrical stimulation (NMES) and sensory electrical stimulation (SES). SES was particularly useful for maintaining or even improving muscle function, control movement, and postural balance in older subjects, provided that their basal functional capabilities are not reduced and their risk of falling is low. In turn, in frail older subjects with diminished basal functional capabilities and at high risk of falling, NMES can potentially boost their neuromuscular system to recondition lower-limb muscle strength/power and thus limit the risk of falling.

The literature search of original data and review by Lefferts et al. summarized the physiological effects of acute exercise on the brain (cognitive, brain-blood-barrier), cardiovascular, neuroendocrine, inflammation/oxidative stress, metabolic, and musculoskeletal systems and then aligned those observations with literature describing changes seen with aging and age-related chronic disease. These authors concluded that regular exercise protects against aging and age-related chronic disease exercise acts as an aging mimetic.

The meta-analysis by Ma et al. evaluated the effect of regular aerobic exercise on renal function in patients with chronic kidney disease. Regular aerobic exercise has significant effects on the estimated glomerular filtration rate, serum creatinine, 24-h urine protein amount, and blood urea nitrogen in patients with chronic kidney disease, and aerobic exercise with a single exercise duration longer than 30 min has a more significant effect on the estimated glomerular filtration rates, and aerobic exercise by walking or running can more effectively improve serum creatinine in patients with chronic kidney disease.

The review by Krüger et al. described the effects of exercise on the treatment of dyslipidemia and discussed possible immunological-related mechanisms. The authors concluded that if statin therapy is indicated, it can be combined with activity and sports programs without any concerns. If patients follow the activity recommendations in the long term, effects on blood lipids, especially on HDL and triglycerides, can occur after a few weeks. However, most patients with lipometabolic disorders have multiple morbidities that are influenced by physical activity. Here, it is particularly important to clarify the patients’ fitness for sports by means of a sports medicine stress examination.

We as editors of this volume are extremely grateful to the authors for their contributions and hard work in bringing forth their scientific research. We hope the readers of these papers can gain insight from them and utilize the information herein to advance their scientific pursuits.
